# Predictors of Genital Hygiene Behaviors Among Women Recruited Through an Online Survey in Türkiye: The Role of Health Literacy

**DOI:** 10.3390/healthcare14183066

**Published:** 2026-09-17

**Authors:** Nigar Çelik, Özlem Güner

**Affiliations:** 1 Department of Obstetrics and Gynecology Nursing, Kutahya Health Sciences University, Kutahya 43100, Türkiye; nigar.celik@ksbu.edu.tr; 2 Department of Midwifery, Faculty of Health Sciences, Sinop University, Sinop 57000, Türkiye

**Keywords:** health literacy, genital hygiene, women’s health, reproductive health, health behavior

## Abstract

**Aim:** This study aimed to determine health literacy and genital hygiene behavior levels, examine the relationship between these variables, and evaluate the factors associated with genital hygiene behaviors among women aged 18 years and older who were recruited through an online survey in Türkiye. **Methods:** This descriptive cross-sectional study was conducted in Türkiye between 31 March and May 2026 with 469 women aged 18 years and older who were recruited through an online survey distributed via social media. Data were collected using the Health Literacy Scale and the Genital Hygiene Behaviors Scale. Descriptive statistics, Mann–Whitney U test, Kruskal–Wallis test, Spearman correlation analysis, and multiple linear regression analysis were used. **Results:** The mean Health Literacy Scale score was 109.61 ± 13.77 (possible range: 25–125), while the mean Genital Hygiene Behaviors Scale score was 95.94 ± 9.91 (possible range: 23–115). A positive, statistically significant, but small correlation was found between health literacy and genital hygiene behaviors (r = 0.292, *p* < 0.001). In the regression analysis, health literacy was independently associated with genital hygiene behaviors (β = 0.344, *p* < 0.001). Receiving genital hygiene education or information (β = 0.142, *p* = 0.001) and consulting a physician when experiencing a health problem (β = 0.094, *p* = 0.019) were also independently associated with genital hygiene behaviors. **Conclusions:** Higher health literacy was associated with more positive genital hygiene behaviors among women recruited online in Türkiye; however, the magnitude of the association was small. Receiving genital hygiene education or information and seeking professional healthcare were also associated with more positive behaviors. These findings support further investigation of health literacy-informed educational approaches, particularly through longitudinal and intervention studies, rather than establishing that such approaches would causally improve genital hygiene behaviors.

## 1. Introduction

Genital hygiene behaviors are among the most fundamental self-care practices for maintaining women’s reproductive health. Due to the anatomical structure of the female genital system, including the close proximity of the urethra, vagina, and anus, as well as physiological processes such as menstruation, sexual activity, perspiration, and genital secretions, women may be more susceptible to urogenital infections. Inappropriate genital hygiene practices may contribute to vaginal discharge, itching, burning, urinary tract infections, and recurrent genital infections. Although these conditions are often not life-threatening, untreated infections may lead to more serious consequences, including pelvic inflammatory disease, infertility, pregnancy complications, and reduced quality of life [1,2,3].

Genital hygiene behaviors cannot be explained solely by individual cleaning habits. Factors such as age, educational level, income status, living environment, family and cultural characteristics, access to healthcare services, knowledge regarding menstrual hygiene, attention to genital symptoms, and the likelihood of seeking advice from healthcare professionals may influence these behaviors. Previous studies have reported that educational level, income status, employment status, receiving genital hygiene education, obtaining information from physicians or nurses, and utilization of healthcare services are associated with genital hygiene behaviors [1,2,3].

Maintaining optimal genital hygiene behaviors requires more than simply possessing knowledge. Women need to be able to access accurate health information, understand it, evaluate its reliability, and apply it to their daily lives. In this context, health literacy has emerged as a key determinant of how women interpret and translate health information into preventive health behaviors and everyday health-related decisions. Higher levels of health literacy have been associated with the adoption of preventive health behaviors, effective use of healthcare services, and health promotion [4,5,6,7].

From the perspective of women’s reproductive health, health literacy encompasses the ability to obtain, interpret, evaluate, and utilize information related to sexual health, family planning, pregnancy, childbirth, the postpartum period, screening programs, and genital health. Evidence from systematic reviews indicates that health literacy is associated with women’s reproductive health knowledge, preventive health behaviors, and utilization of healthcare services [7]. Furthermore, qualitative studies have shown that women perceive reproductive health literacy not merely as possessing knowledge but also as the ability to identify reliable information sources, consult healthcare professionals, and translate acquired information into daily health practices [6].

Although studies directly examining the relationship between health literacy and genital hygiene behaviors are limited, existing evidence suggests that women with higher levels of health literacy tend to exhibit more positive genital hygiene behaviors. A study conducted among female university students in Türkiye reported a significant positive association between health literacy and genital hygiene behaviors and emphasized the importance of access to reliable health information for adopting appropriate hygiene practices [8]. Similarly, women who had received education or counseling regarding genital hygiene were reported to have higher genital hygiene behavior scores [2,3]. These findings suggest that genital hygiene behaviors may be amenable to educational and supportive approaches; however, their effectiveness should be evaluated in intervention studies.

Despite the existence of studies examining factors associated with genital hygiene behaviors in Türkiye, research evaluating the relationship between general health literacy and genital hygiene behaviors among women outside narrowly defined clinical populations remains limited. Health literacy is considered an important determinant that may influence women’s ability to recognize health problems, seek appropriate healthcare, obtain information from healthcare professionals, and maintain preventive health behaviors. Therefore, investigating the relationship between health literacy and genital hygiene behaviors may contribute to the planning of educational and counseling programs aimed at improving women’s health, strengthening preventive healthcare services, and promoting reproductive health.

This study was conducted to determine health literacy and genital hygiene behavior levels, examine the relationship between health literacy and genital hygiene behaviors, and evaluate the factors associated with genital hygiene behaviors among women aged 18 years and older who were recruited through an online survey in Türkiye. The findings may contribute to the development of future genital hygiene education and health literacy interventions tailored to digitally reachable populations, while providing preliminary evidence for more representative population-based studies.

### Research Questions

1. What are the levels of health literacy and genital hygiene behaviors among women?
2. Is there a relationship between women’s health literacy levels and genital hygiene behaviors?
3. What factors are associated with women’s genital hygiene behaviors?

## 2. Methods

### 2.1. Study Design

This descriptive cross-sectional study was conducted to determine women’s health literacy and genital hygiene behavior levels, examine the relationship between health literacy and genital hygiene behaviors, and evaluate factors associated with genital hygiene behaviors.

### 2.2. Population and Sample

The intended target population comprised women aged 18 years and older living in Türkiye; the study was not specifically designed to include only women in the university education category. However, the achieved sample consisted of women who encountered and responded to the online survey distributed through social media during the study period, agreed to participate, and met the eligibility criteria. Convenience sampling, a non-probability sampling method, was used. Cochran’s formula was used to establish an initial recruitment target of 384 participants, based on a 95% confidence level, an assumed prevalence of 50%, and a nominal 5% margin of error. However, because no sampling frame or random selection procedure was used, the theoretical margin of error associated with simple random sampling does not apply to the achieved sample. A total of 469 women were included in the final analysis. Exceeding the initial recruitment target increased the number of observations available for analysis but did not make the sample representative of all women living in Türkiye. Inclusion Criteria: Women were eligible to participate if they were aged 18 years or older, living in Türkiye, able to read and understand Turkish, provided electronic informed consent, and completed the primary study instruments.

### 2.3. Exclusion Criteria

Individuals who declined participation, were younger than 18 years, were unable to read and understand Turkish, or did not complete the primary study instruments were excluded. Participants with an isolated missing response to a sociodemographic item were retained, and analyses involving that variable were conducted using available cases.

### 2.4. Data Collection Instruments

Data were collected using an online questionnaire consisting of three sections. The first section included the Descriptive Information Form developed by the researchers based on the relevant literature, the second section included the Health Literacy Scale, and the third section included the Genital Hygiene Behaviors Scale.

### 2.5. Descriptive Information Form

The Descriptive Information Form was developed by the researchers based on the relevant literature to assess participants’ sociodemographic and health-related characteristics [1,2,3,8,9]. The form consisted of 24 questions covering age, height, weight, native language, marital status, family type, employment status, educational level, income level, place of residence, smoking and alcohol use, reproductive characteristics, history of gynecological examination or Pap smear testing, history of diseases related to female reproductive organs, receipt of genital hygiene education or information, health-seeking behaviors, sources of health information, perceived health status, physical exercise, and contraceptive use.

### 2.6. Health Literacy Scale

The multidimensional framework underlying the Health Literacy Scale was described by Sørensen et al. [5]. The 25-item HLS used in the present study was developed by Toçi et al. [10], and its Turkish validity and reliability were established by Aras and Bayık Temel [11]. The scale consists of four subdimensions: Access to Information, Understanding Information, Appraisal/Evaluation, and Application/Use. Responses are rated on a five-point Likert scale ranging from 1 (“unable to do/no ability/impossible”) to 5 (“no difficulty”). There are no reverse-scored items. Total scores range from 25 to 125, with higher scores indicating higher levels of health literacy. The original study reported a Cronbach’s alpha coefficient of 0.92 [11]. In the present study, Cronbach’s alpha was 0.952.

### 2.7. Genital Hygiene Behaviors Scale

The Genital Hygiene Behaviors Scale was developed and validated by Karahan [12]. The scale consists of 23 items and three subdimensions: general hygiene habits, menstrual hygiene, and awareness of abnormal findings. The first 12 items assess general hygiene habits, items 13–20 assess menstrual hygiene, and items 21–23 assess awareness of abnormal findings. Responses are rated on a five-point Likert scale ranging from “strongly agree” to “strongly disagree.” Items 7, 14, 19, 20, and 23 are reverse scored. Total scores range from 23 to 115, with higher scores indicating more positive genital hygiene behaviors. In the original study, Cronbach’s alpha coefficients were reported as 0.80 for the total scale, 0.70 for general hygiene habits, 0.74 for menstrual hygiene, and 0.81 for awareness of abnormal findings [12]. In the present study, Cronbach’s alpha for the total scale was 0.793.

### 2.8. Data Collection Procedure

Data were collected between 31 March and May 2026 using a self-administered online survey created with Google Forms. The questionnaire link was distributed through Facebook, Instagram, WhatsApp, and X. Recruitment was therefore limited to women who used these platforms, encountered the shared survey link, and voluntarily chose to participate. The social media platform through which each participant accessed the questionnaire was not recorded; therefore, platform-specific recruitment and completion rates could not be determined. Upon accessing the survey link, participants first read the informed consent form. Only those who provided electronic informed consent were directed to the questionnaire. No names, identification numbers, IP addresses, or other directly identifying information were collected, and all responses were obtained anonymously. Participants were informed that participation was voluntary and that they could withdraw before submitting the questionnaire without penalty. No financial or non-financial incentives were provided. Completion of the questionnaire required approximately 10–15 min. A total of 469 questionnaires were submitted. All submitted questionnaires contained complete responses to the primary study instruments—the HLS and GHBS—and, therefore, none were excluded because of incomplete primary study data. All 469 questionnaires were included in the main analyses, corresponding to a 100% retention rate among submitted questionnaires. Isolated missing responses to sociodemographic items were retained, and analyses involving those variables were conducted using available cases without imputation. The number of individuals who viewed or opened the survey link without submitting the questionnaire was not recorded; therefore, a view-based participation rate could not be calculated. Data confidentiality was maintained through a password-protected Google Forms account accessible only to the research team. No formal cognitive screening, researcher-administered assessment, or separate screening question was used because the survey was self-administered online. Participants’ ability to understand and complete the questionnaire was inferred from their provision of electronic informed consent and successful completion of the primary study instruments.

### 2.9. Data Analysis

Data were analyzed using IBM SPSS Statistics version 26 (IBM Corp., Armonk, NY, USA). Descriptive statistics were presented as frequencies and percentages for categorical variables. For continuous variables, both mean ± standard deviation and median with interquartile range were reported to provide complementary descriptive information and facilitate comparison with previous scale-based studies; observed minimum and maximum values were also presented where appropriate. Because the scale and subscale scores were not normally distributed, medians and interquartile ranges were used as the primary distribution-based summaries, and non-parametric tests were used for group comparisons and correlation analyses.

The normality of scale and subscale scores was assessed using the Shapiro–Wilk test, Kolmogorov–Smirnov test, skewness and kurtosis values, and measures of central tendency. Since the data were not normally distributed, the Mann–Whitney U test was used for comparisons between two groups, and the Kruskal–Wallis test was used for comparisons among three or more groups. When significant differences were identified with the Kruskal–Wallis test, Bonferroni-adjusted pairwise comparisons were performed.

The relationships between the total and subscale scores of the Health Literacy Scale and the Genital Hygiene Behaviors Scale were examined using Spearman correlation analysis. To identify factors associated with genital hygiene behaviors, multiple linear regression analysis was performed using the total Genital Hygiene Behaviors Scale score as the dependent variable. Health literacy scores and variables found to be significant in univariate analyses or considered theoretically relevant were included in the model.

Prior to regression analysis, the assumptions of linear regression were evaluated. Multicollinearity was assessed using tolerance and Variance Inflation Factor (VIF) values, and no serious multicollinearity problems were detected. Regression coefficients were estimated using ordinary least squares (OLS), and standard errors were calculated using the heteroscedasticity-consistent HC3 robust covariance estimator. Internal consistency of the scales was evaluated using Cronbach’s alpha coefficients. Statistical significance was set at *p* < 0.05. Missing data were not imputed. Analyses involving childbirth history were performed using the 395 participants with available data for this variable.

### 2.10. Ethical Considerations

Ethical approval was obtained from the Sinop University Human Research Ethics Committee (Decision No. 2026/127, dated 31 March 2026). Permission to use the scales was obtained from the respective copyright holders via e-mail. Before beginning the online questionnaire, all participants were informed about the purpose and scope of the study, the voluntary nature of participation, their right to withdraw at any time, the confidentiality and anonymity of the collected data, and the use of the data solely for scientific purposes. Electronic informed consent was obtained by requiring participants to read the informed consent statement and select the “I agree to participate in the study” option before proceeding to the questionnaire. No names, IP addresses, or other directly identifying information were collected, and all data were obtained anonymously. The study was conducted in accordance with the Declaration of Helsinki and reported following the STROBE guidelines.

## 3. Results

A total of 469 women participated in the study. The majority of the participants were aged 24 years or younger (65.9%), single (69.7%), living in a nuclear family (91.3%), and classified in the university education category (79.7%). Among the participants, 53.5% reported that their income did not cover their expenses, 72.7% lived in a district/town/village, 67.2% had never smoked, and 69.7% had never consumed alcohol. Additionally, 29.0% had previously undergone a gynecological examination or Pap smear test, 15.8% reported a history of reproductive organ disease, and 47.8% had received education or information regarding genital hygiene. The proportion of women who reported consulting a physician when experiencing a health problem was 80.4% (Table 1). Childbirth history data were available for 395 participants; 74 participants did not respond to this item.

Comparison of Health Literacy Scale (HLS) total scores according to participants’ characteristics revealed statistically significant differences by age group, income status, place of residence, and alcohol use (*p* < 0.05). Examination of median scores showed that HLS scores were higher among women aged 25–34 years and ≥35 years, those living in districts/towns/villages, and those who currently or previously consumed alcohol. No significant differences were found in HLS total scores according to marital status, employment status, family type, educational level, smoking status, childbirth history, history of gynecological examination/Pap smear, history of reproductive organ disease, receiving genital hygiene education/information, health-seeking behavior, place of health check-up, perceived health status, exercise status, or contraceptive use (*p* > 0.05) (Table 1).

The total score on the Genital Hygiene Behaviors Scale (GHBS) differed significantly according to age group, place of residence, smoking status, alcohol use, history of gynecological examination/Pap smear, receipt of genital hygiene education/information, health-seeking behavior, and exercise status (*p* < 0.05). Median scores indicated that GHBS scores were higher among older participants, those living in districts/towns/villages, current smokers, women who currently or previously consumed alcohol, those who had undergone a gynecological examination/Pap smear, those who had received education or information on genital hygiene, women who consulted a physician when experiencing a health problem, and those who exercised regularly. In contrast, no significant differences in GHBS total scores were observed according to marital status, employment status, family type, educational level, income status, childbirth history, history of reproductive organ disease, place of health check-up, perceived health status, or contraceptive use (*p* > 0.05) (Table 1).

The mean total Genital Hygiene Behaviors Scale score was 95.94 ± 9.91, with a median score of 97.00 (90.00–103.00). The mean subscale scores were 49.69 ± 5.73 for General Hygiene Habits, 34.31 ± 4.20 for Menstrual Hygiene, and 11.94 ± 2.43 for Awareness of Abnormal Findings. Cronbach’s alpha was 0.793 for the total GHBS, 0.704 for General Hygiene Habits, 0.624 for Menstrual Hygiene, and 0.470 for Awareness of Abnormal Findings (Table 2). The two latter subscales were therefore interpreted cautiously in the subsequent analyses. Item-level descriptive statistics for the HLS and GHBS are presented in Supplementary Table S1.

**Table 2:** Total and Subscale Scores of the Health Literacy Scale and Genital Hygiene Behaviors Scale.

Scale and Subscales	Possible Score Range	Observed Min–Max	Mean ± SD	Median (Q1–Q3)	Cronbach α
Health Literacy Scale	25–125	25–125	109.61 ± 13.77	112.00 (100.00–121.00)	0.952
Access to information	5–25	5–25	22.49 ± 3.02	24.00 (20.00–25.00)	0.902
Understanding information	7–35	7–35	30.83 ± 4.22	32.00 (28.00–35.00)	0.862
Appraisal/Evaluation	8–40	8–40	35.01 ± 4.95	36.00 (32.00–39.00)	0.901
Application/Use	5–25	5–25	21.28 ± 3.36	22.00 (19.00–25.00)	0.820
Genital Hygiene Behaviors Scale	23–115	43–114	95.94 ± 9.91	97.00 (90.00–103.00)	0.793
General hygiene habits	12–60	16–60	49.69 ± 5.73	51.00 (47.00–54.00)	0.704
Menstrual hygiene	8–40	20–40	34.31 ± 4.20	35.00 (31.00–38.00)	0.624
Awareness of abnormal findings	3–15	3–15	11.94 ± 2.43	12.00 (11.00–14.00)	0.470

Note. SD = standard deviation; Q1–Q3 = 25th–75th percentiles. Both mean ± SD and median (Q1–Q3) are presented for descriptive purposes. Because the scale and subscale scores were not normally distributed, median (Q1–Q3) was considered the primary distribution-based summary, and non-parametric methods were used for inferential analyses. Higher Health Literacy Scale scores indicate higher health literacy, whereas higher Genital Hygiene Behaviors Scale scores indicate more positive genital hygiene behaviors. Items 7, 14, 19, 20, and 23 of the Genital Hygiene Behaviors Scale were reverse-scored. The relationships between HLS total and subscale scores and GHBS total and subscale scores were examined using Spearman correlation analysis. A positive, statistically significant, but small correlation was found between the HLS total score and the GHBS total score (r = 0.292, *p* < 0.001), indicating that higher health literacy was associated with more positive genital hygiene behaviors, although the magnitude of the association was modest. The HLS total score was also positively correlated with the GHBS subscales of General Hygiene Habits (r = 0.238, *p* < 0.001), Menstrual Hygiene (r = 0.278, *p* < 0.001), and Awareness of Abnormal Findings (r = 0.175, *p* < 0.001). Among the HLS subscales, the strongest correlation with the GHBS total score was observed for the Appraisal/Evaluation subscale (r = 0.278, *p* < 0.001), followed by Access to Information (r = 0.268, *p* < 0.001), Understanding Information (r = 0.255, *p* < 0.001), and Application/Use (r = 0.227, *p* < 0.001) (Table 3).

**Table 3:** Correlations Between Health Literacy Scale and Genital Hygiene Behaviors Scale Scores.

HLS Total and Subscales	GHBS General Hygiene Habits	GHBS Menstrual Hygiene	GHBS Awareness of Abnormal Findings	GHBS Total
Access to information	r = 0.203, *p* < 0.001	r = 0.279, *p* < 0.001	r = 0.152, *p* = 0.001	r = 0.268, *p* < 0.001
Understanding information	r = 0.212, *p* < 0.001	r = 0.231, *p* < 0.001	r = 0.149, *p* = 0.001	r = 0.255, *p* < 0.001
Appraisal/Evaluation	r = 0.220, *p* < 0.001	r = 0.269, *p* < 0.001	r = 0.172, *p* < 0.001	r = 0.278, *p* < 0.001
Application/Use	r = 0.196, *p* < 0.001	r = 0.212, *p* < 0.001	r = 0.150, *p* = 0.001	r = 0.227, *p* < 0.001
HLS total	r = 0.238, *p* < 0.001	r = 0.278, *p* < 0.001	r = 0.175, *p* < 0.001	r = 0.292, *p* < 0.001

Note. HLS = Health Literacy Scale; GHBS = Genital Hygiene Behaviors Scale. Correlations were evaluated using Spearman correlation analysis. r = Spearman correlation coefficient.

To identify factors associated with genital hygiene behaviors, a multiple linear regression analysis was performed with the GHBS total score as the dependent variable. Health literacy total score, age, place of residence, smoking status, alcohol use, history of gynecological examination/Pap smear, receiving genital hygiene education/information, consulting a physician when experiencing a health problem, and exercise status were entered into the model. The overall model was statistically significant and explained approximately 19.6% of the variance in GHBS total scores (R^2^ = 0.196, adjusted R^2^ = 0.178, F = 7.431, *p* < 0.001).

Health literacy had the largest standardized regression coefficient among the variables included in the model (β = 0.344, *p* < 0.001). Higher HLS scores were associated with higher GHBS total scores (B = 0.248, β = 0.344, *p* < 0.001). Furthermore, receiving genital hygiene education or information (B = 2.816, β = 0.142, *p* = 0.001) and consulting a physician when experiencing a health problem (B = 2.337, β = 0.094, *p* = 0.019) were significantly and independently associated with higher GHBS total scores. Although age, place of residence, smoking status, alcohol use, history of gynecological examination/Pap smear, and exercise status showed significant associations in the univariate analyses, they were not identified as independent predictors of GHBS total scores in the multivariable model (*p* > 0.05) (Table 4).

## 4. Discussion

The main finding of this study was a statistically significant but small positive association between health literacy and genital hygiene behaviors among the participants. Higher total HLS scores were associated with higher total GHBS scores (r = 0.292, *p* < 0.001), although the magnitude of this relationship was modest. Furthermore, health literacy had the largest standardized regression coefficient among the variables included in the multiple regression model. These findings support considering genital hygiene behaviors not solely as routine hygiene practices but also as women’s health behaviors that may be associated with the ability to access, understand, evaluate, and apply health-related information.

The characteristics of the achieved sample should be considered when interpreting the relatively high HLS and GHBS scores. Although the study was intended to reach adult women living in Türkiye rather than exclusively women in the university education category, women aged 24 years or younger (65.9%) and those in the university education category (79.7%) were overrepresented in the achieved sample. Recruitment through social media may have contributed to the overrepresentation of younger, more highly educated, and digitally connected women, consistent with previously identified inequalities in access to and engagement with digital health resources [13,14]. Higher educational attainment and greater digital access may facilitate access to, comprehension of, and engagement with health information and may therefore have contributed to the relatively high HLS and GHBS scores observed in this sample [13,14]. Accordingly, the observed score levels and associations should be interpreted as findings from the achieved online sample rather than as population estimates for all women living in Türkiye. The participants’ fields of education were not recorded; therefore, the proportion of participants with education in health-related disciplines could not be determined. Formal education in medicine, nursing, midwifery, nutrition, or another health-related field may have influenced both HLS and GHBS scores and may partly account for the observed association between the two measures. This finding is consistent with the study conducted by Sağlık et al. (2024), which directly examined the relationship between health literacy and genital hygiene behaviors [8]. In their study of female university students, a significant positive association was reported between health literacy and genital hygiene behavior scores, indicating that higher levels of health literacy were associated with more favorable genital hygiene behaviors. The authors also emphasized the importance of access to reliable health information for learning appropriate genital hygiene practices. In the present study, health literacy remained significantly associated with genital hygiene behaviors after adjustment for the other variables included in the model. However, this finding does not establish causality or exclude residual confounding.

Comparable findings have also been reported in the broader reproductive health literacy literature. In an online cross-sectional study of 386 women of reproductive age in Vienna, Rottjakob et al. (2026) found that inadequate health literacy was associated with lower reproductive health knowledge and behavior scores; however, this association weakened after reproductive health information sources were included in the model [15]. This finding supports the relevance of health literacy while also indicating that the sources from which women obtain health information may contribute to the observed relationship. Similarly, Chawłowska et al. (2020) reported incomplete reproductive health and fertility knowledge among 456 Polish female university students, despite the educational profile of the sample, with better knowledge observed among medical university students [16]. This finding is particularly relevant to the present study because participants’ fields of education were not recorded, and health-related education may have influenced both health literacy and genital hygiene behavior scores. Collectively, these studies suggest that education level alone may not ensure adequate reproductive health knowledge or behavior and that health literacy, educational field, and information sources should be considered together when interpreting women’s reproductive health behaviors.

The theoretical basis of this relationship is also supported by the reproductive health literacy literature [6,7,13]. Bakht et al. (2023) defined women’s reproductive health literacy not merely as possessing information but as the ability to access, understand, evaluate, and use reproductive health information to promote health-related behaviors [6]. Similarly, the systematic review by Kilfoyle et al. (2016) reported that health literacy is associated with women’s reproductive health knowledge and various preventive health behaviors [7]. Accordingly, women with higher health literacy may be better equipped to distinguish appropriate from inappropriate genital hygiene practices, recognize abnormal symptoms, and seek professional healthcare when necessary.

The reliability findings should be considered when interpreting the subscale-level associations. Although the internal consistency of the total GHBS was acceptable (α = 0.793), the coefficients for Menstrual Hygiene (α = 0.624) and particularly Awareness of Abnormal Findings (α = 0.470) were below the commonly used threshold of 0.70 [17]. The Awareness of Abnormal Findings subscale consisted of only three items; because Cronbach’s alpha is influenced by both the number of items and the correlations among those items, the limited number of items may have contributed to the low coefficient [17,18]. Consequently, associations involving these two subscales, particularly Awareness of Abnormal Findings, should be interpreted cautiously. Measurement unreliability may attenuate observed correlations; therefore, the consistently weaker correlations between Awareness of Abnormal Findings and the HLS domains may partly reflect limited measurement reliability rather than an absence of an underlying relationship [18]. Importantly, the multiple regression analysis used the total GHBS score rather than the less reliable subscale scores as the dependent variable.

The magnitude of the observed associations also warrants careful interpretation. According to Cohen’s conventional benchmarks, all correlations between the HLS and GHBS total and subscale scores were within the small-effect range (r = 0.149–0.292) [19]. Thus, although these correlations were statistically significant, their practical magnitude was modest, and statistical significance may have been facilitated by the relatively large sample size. Among the HLS domains, Appraisal/Evaluation showed the strongest correlation with the total GHBS score (r = 0.278), suggesting that the ability to critically assess health information may be a particularly relevant area for future intervention research. In contrast, Awareness of Abnormal Findings showed the weakest correlations with all HLS domains (r = 0.149–0.172); however, this pattern should be interpreted in light of the low internal consistency of this three-item subscale. Furthermore, the multivariable model explained 19.6% of the variance in total GHBS scores (R^2^ = 0.196), indicating that most of the variance remained unexplained by the model. Health literacy should therefore be considered one relevant correlate of genital hygiene behaviors rather than a comprehensive explanation of these behaviors.

In the present study, both Health Literacy Scale and Genital Hygiene Behaviors Scale scores were generally high. Although the mean scores reported by Sağlık et al. (2024) among female university students were lower than those observed in our study, both studies demonstrated favorable levels of health literacy and genital hygiene behaviors and identified a significant relationship between these variables [8]. Likewise, Karahan et al. (2025) reported a mean GHBS score of 94.93 ± 9.9 in a Turkish sample, which was comparable to the score observed in our study [9]. In contrast, Abiç (2025) reported lower genital hygiene behavior scores among women of reproductive age [3]. These differences may be attributable to variations in age, educational background, data collection methods, healthcare access, and exposure to genital hygiene information across study populations.

Another important finding was that women who had received education or information regarding genital hygiene demonstrated higher genital hygiene behavior scores. Moreover, receiving genital hygiene education or information remained independently associated with GHBS scores in the multivariable model. This finding is consistent with Abiç (2025), who reported higher genital hygiene behavior scores among women who had received genital hygiene education [3]. Similarly, Karahan et al. (2025) found significantly higher scores among women who had received such education compared with those who had not [9]. Çankaya and Dereli Yılmaz (2015) also reported a significant association between receiving education on genital hygiene and genital hygiene behaviors, emphasizing the importance of educational interventions [1].

Studies examining educational interventions further support these findings. Gökşin and Demirhan (2024) reported significant improvements in total GHBS scores following genital hygiene education among adolescent girls. Increases were also observed in subdimensions related to general hygiene, menstrual hygiene, and awareness of abnormal symptoms [20]. Likewise, Çetinkaya Ak et al. (2026) identified genital hygiene education as a positive predictor of genital hygiene behaviors among female university students [21]. Collectively, these findings suggest that genital hygiene education may be associated not only with improved knowledge but also with behavioral domains such as appropriate perineal hygiene, menstrual hygiene practices, and recognition of abnormal symptoms.

Women who reported consulting a physician when experiencing health problems had significantly higher genital hygiene behavior scores, and this variable remained a significant predictor in the regression model. In addition, women with a history of gynecological examination or Pap smear testing demonstrated higher GHBS scores in univariate analyses. Uzun et al. (2022) similarly found higher genital hygiene behavior scores among women who reported obtaining genital hygiene information from healthcare professionals [2]. Maricic et al. (2021) reported that women with adequate health literacy demonstrated more favorable reproductive health behaviors, including gynecological follow-up, Pap testing, and active engagement with health-related information [22]. These findings suggest that contact with healthcare services may facilitate not only healthcare utilization but also access to reliable health information and the adoption of healthier behaviors.

Age, place of residence, smoking status, alcohol use, history of gynecological examination or Pap smear testing, and exercise status were significantly associated with GHBS scores in the bivariate analyses presented in Table 1. Previous studies have similarly reported variations in genital hygiene behaviors according to sociodemographic, lifestyle, educational, and healthcare-related characteristics [1,2,3,9,23,24]. However, these variables were no longer statistically significant after health literacy and the other covariates were considered simultaneously in the multivariable model presented in Table 4. This attenuation suggests that their unadjusted associations with genital hygiene behaviors were not independent of the other variables included in the model. The observed differences may partly reflect variation in health literacy, educational characteristics, access to health information, or healthcare utilization across these groups rather than direct effects of age, residence, smoking, or alcohol use on genital hygiene behaviors. Alternatively, some of these characteristics may be associated with genital hygiene behaviors through pathways involving health literacy. However, because no formal mediation analysis was performed and the data were cross-sectional, mediation cannot be established or distinguished from confounding. Accordingly, the bivariate associations should be interpreted cautiously and should not be considered evidence of independent or causal effects.

The regression model explained approximately 19.6% of the variance in genital hygiene behaviors. Although health literacy emerged as an important factor, this finding indicates that genital hygiene behaviors cannot be explained solely by health literacy. Such behaviors are multidimensional and may be influenced by individual knowledge and skills as well as cultural norms, health beliefs, family characteristics, environmental conditions, economic resources, and access to healthcare services [9]. Future studies incorporating these factors may provide a more comprehensive understanding of genital hygiene behaviors.

Genital hygiene behaviors should not be viewed solely as hygiene-related practices. Access to reliable information is essential for maintaining genital health [25,26]. Almuhayya et al. (2026) reported that women’s knowledge regarding the vaginal microbiome may be limited and that certain hygiene practices may negatively affect vaginal health [27]. Similarly, Binmahfoodh et al. (2026) found that women frequently use social media and other informal sources, in addition to healthcare professionals, when seeking information about genital hygiene [28]. These findings highlight the importance of enabling women to critically evaluate health information and distinguish reliable sources from misinformation.

From a public health perspective, the observed associations suggest that genital hygiene behaviors should be considered within the broader context of access to, understanding of, and critical engagement with health information [13]. Although the effect sizes were small and the cross-sectional design does not establish causality, the findings identify potentially modifiable areas that warrant investigation in future health promotion programs. Previous research indicating that genital hygiene education is associated with more positive hygiene behaviors supports further evaluation of integrating genital and menstrual health information into primary healthcare counseling and community-based education [2,3,20]. Such initiatives could address inappropriate hygiene practices, support timely recognition of abnormal symptoms, and encourage appropriate healthcare-seeking behavior [25,28]. However, reliance on digital education alone may widen existing inequalities because older adults and individuals with lower educational attainment, limited digital literacy, or restricted internet access may experience barriers to digital health interventions [14]. Therefore, combining accessible digital resources with face-to-face counseling, community outreach, and human support may provide a more equitable approach [14]. Future programs should be culturally appropriate, understandable across different literacy levels, and evaluated in terms of both behavioral outcomes and equitable reach. This study contributes to the literature by examining health literacy and genital hygiene behaviors together and by evaluating potential factors associated with genital hygiene behaviors using a multivariable approach. The findings support the development and evaluation of health promotion programs that extend beyond the general provision of genital hygiene information. Such programs could combine health literacy training with practical counseling on evidence-based genital and menstrual hygiene practices, recognition of abnormal symptoms, identification of reliable information sources, and appropriate use of primary healthcare services. To reach women who were underrepresented in the present online sample, these programs could be delivered through family health centers, community health services, women’s health clinics, municipal programs, and outreach activities, in addition to digital platforms. Their effectiveness should be evaluated using predefined and measurable outcomes, including changes in mean GHBS scores, the proportion of women reporting evidence-based hygiene practices, recognition of symptoms requiring professional evaluation, and appropriate healthcare-seeking behavior. Because the GHBS has no established clinical cut-off score defining adequate behavior, the present findings do not support the specification of an arbitrary target percentage; intervention studies are needed to determine meaningful and achievable levels of improvement.

## 5. Limitations

This study has several limitations. First, its cross-sectional design precludes causal inferences regarding the relationship between health literacy and genital hygiene behaviors. Second, because the data were collected using self-report measures, recall and social desirability biases may have influenced participants’ responses. Third, recruitment through social media using convenience sampling introduced both selection and coverage bias. Women without internet or smartphone access, those with limited digital literacy, and those who did not use the selected social media platforms had little or no opportunity to participate. Consequently, younger women and women in the university education category were overrepresented in the sample. Their potentially greater access to and familiarity with health information may have contributed to the relatively high HLS and GHBS scores. Moreover, because no sampling frame or random selection procedure was used, the nominal 5% margin of error derived from Cochran’s formula does not apply strictly to the achieved sample, and the sample size alone does not ensure representativeness. Therefore, the findings cannot be generalized to all women living in Türkiye, particularly older women, women with lower educational attainment or limited digital literacy, and those from socioeconomically disadvantaged or digitally underserved groups. In addition, participants’ fields of education were not collected. Consequently, health-related educational background could not be included as a covariate in the regression model. This unmeasured factor may have confounded the observed association between health literacy and genital hygiene behaviors because formal health education could independently contribute to higher scores on both measures. Finally, the internal consistency coefficients for the Menstrual Hygiene (α = 0.624) and Awareness of Abnormal Findings (α = 0.470) subscales were below the conventional threshold of 0.70. Therefore, findings involving these subscales, particularly the three-item Awareness of Abnormal Findings subscale, should be interpreted cautiously.

## 6. Conclusions and Recommendations

This study identified a statistically significant but small positive association between health literacy and genital hygiene behaviors among women recruited through an online survey in Türkiye. Health literacy had the largest standardized regression coefficient among the variables included in the model. Receiving genital hygiene education or information and consulting a physician when experiencing a health problem were also independently associated with higher GHBS total scores. However, because of the cross-sectional design, these findings do not demonstrate that improving health literacy or providing education would causally improve genital hygiene behaviors.

The findings identify health literacy and genital hygiene education as potentially relevant areas for further investigation. Future intervention studies could evaluate whether programs combining evidence-based genital and menstrual hygiene information with skills for accessing and critically evaluating health information produce meaningful improvements in GHBS scores and specific hygiene practices. Recommendations concerning the recognition of abnormal genital findings should be interpreted particularly cautiously because the relevant three-item subscale demonstrated low internal consistency in the present sample.

Future studies should recruit women from more diverse age, educational, socioeconomic, and digital-access groups and use probability-based sampling where feasible. Longitudinal and experimental studies are needed to clarify the direction of the observed associations and to determine whether changes in health literacy are accompanied by improvements in genital hygiene behaviors.

## Figures and Tables

**Table 1:** Comparison of Health Literacy Scale and Genital Hygiene Behaviors Scale Total Scores According to Participants’ Characteristics.

Variables	*n* (%)	HLS Total Median (Q1–Q3)	*p*	GHBS Total Median (Q1–Q3)	*p*
Age group			0.046		0.032
≤24 years	309 (65.9)	110 (100–120)		96 (90–102)	
25–34 years	38 (8.1)	116 (106–122)		98 (93–104)	
≥35 years	122 (26.0)	115 (106–121)		99 (91–105)	
Marital status			0.117		0.203
Married	134 (28.6)	115 (106–121)		98 (91–105)	
Single	327 (69.7)	111 (100–120)		96 (90–102)	
Divorced	8 (1.7)	112 (109–121)		98 (94–104)	
Employment status			0.590		0.178
Employed	101 (21.6)	112 (104–121)		98 (91–104)	
Unemployed	368 (78.4)	112 (100–121)		96 (90–103)	
Family type			0.148		0.130
Nuclear family	428 (91.3)	113 (100–121)		97 (91–103)	
Extended family	41 (8.7)	110 (101–117)		95 (88–101)	
Educational level			0.917		0.524
High school or below	57 (12.2)	112 (104–122)		98 (90–104)	
University	374 (79.7)	112 (100–121)		97 (90–103)	
Postgraduate	38 (8.1)	113 (104–120)		98 (92–105)	
Income status			0.021		0.596
Income does not meet expenses	251 (53.5)	114 (103–122)		98 (91–104)	
Income partially meets expenses	130 (27.7)	110 (99–117)		96 (91–103)	
Income meets expenses	64 (13.6)	113 (101–121)		94 (88–102)	
Income exceeds expenses	24 (5.1)	110 (100–121)		96 (92–104)	
Place of residence			0.026		0.044
City	128 (27.3)	110 (99–120)		96 (90–101)	
District/town/village	341 (72.7)	114 (103–121)		97 (91–104)	
Smoking status			0.053		0.001
Never smoked	315 (67.2)	111 (100–121)		96 (89–102)	
Former smoker	50 (10.7)	109 (101–121)		96 (91–102)	
Current smoker	104 (22.2)	115 (108–120)		99 (94–106)	
Alcohol use			0.014		<0.001
Never used	327 (69.7)	110 (100–121)		96 (90–102)	
Current/former user	142 (30.3)	115 (106–121)		100 (93–105)	
Childbirth history (valid *n* = 395)			0.150		0.399
No	269 (68.1)	112 (100–121)		98 (91–103)	
Yes	126 (31.9)	115 (106–121)		98 (90–104)	
History of gynecological examination/Pap smear			0.085		0.002
Yes	136 (29.0)	115 (106–120)		98 (92–105)	
No	333 (71.0)	111 (100–121)		96 (89–102)	
History of reproductive organ disease			0.941		0.320
Yes	74 (15.8)	113 (105–118)		98 (92–104)	
No	395 (84.2)	112 (100–121)		97 (90–103)	
Received genital hygiene education/information			0.441		<0.001
Yes	224 (47.8)	113 (101–121)		98 (92–104)	
No	245 (52.2)	112 (100–121)		95 (89–102)	
Behavior when experiencing a health problem			0.379		0.009
Ignore it	31 (6.6)	110 (100–116)		94 (87–100)	
Try self-treatment	61 (13.0)	114 (100–119)		95 (89–101)	
Consult a physician	377 (80.4)	113 (101–121)		98 (91–104)	
Place attended for health check-ups			0.791		0.260
Family health center	71 (15.1)	114 (104–120)		95 (89–102)	
Hospital	343 (73.1)	111 (100–121)		98 (91–104)	
Private hospital/clinic	50 (10.7)	112 (102–119)		96 (92–104)	
Do not attend	5 (1.1)	120 (117–121)		96 (88–96)	
Perceived health status			0.103		0.160
Very good/good	293 (62.5)	113 (103–121)		98 (91–103)	
Moderate	162 (34.5)	110 (99–120)		95 (90–102)	
Poor/very poor	14 (3.0)	114 (108–121)		98 (93–106)	
Exercise status			0.892		0.046
Never exercise	143 (30.5)	111 (100–121)		96 (90–102)	
Exercise irregularly	269 (57.4)	111 (100–121)		97 (91–103)	
Exercise regularly	57 (12.2)	114 (104–119)		101 (92–106)	
Contraceptive use			0.227		0.265
No	374 (79.7)	111 (100–121)		97 (90–103)	
Yes	95 (20.3)	115 (106–120)		98 (92–104)	

Note: Data are presented as *n* (%) and median (Q1–Q3). HLS: Health Literacy Scale; GHBS: Genital Hygiene Behaviors Scale. The Mann–Whitney U test was used for comparisons between two groups, and the Kruskal–Wallis test was used for comparisons among three or more groups. Childbirth history data were available for 395 participants; 74 participants did not respond to this item. Percentages for childbirth history were calculated based on valid responses. No imputation was performed, and analyses involving childbirth history were conducted using available cases. A *p*-value < 0.05 was considered statistically significant. The mean total Health Literacy Scale score was 109.61 ± 13.77, with a median score of 112.00 (100.00–121.00). The mean subscale scores were 22.49 ± 3.02 for Access to Information, 30.83 ± 4.22 for Understanding Information, 35.01 ± 4.95 for Appraisal/Evaluation, and 21.28 ± 3.36 for Application/Use. The Cronbach’s alpha coefficient of the HLS in the present study was 0.952.

**Table 4:** Predictors of Total Genital Hygiene Behaviors Scale Score.

Independent Variables	B	SE	95% CI	β	*p*
Constant	65.878	5.055	55.971–75.784	—	<0.001
HLS total score	0.248	0.047	0.155–0.340	0.344	<0.001
Age	−0.072	0.060	−0.190–0.047	−0.077	0.236
Living in district/town/village	0.640	1.098	−1.513–2.793	0.029	0.560
Current smoking	1.902	1.075	−0.206–4.010	0.080	0.077
Alcohol use/history	0.632	1.024	−1.375–2.640	0.029	0.537
Gynecological examination/Pap smear history	2.359	1.327	−0.241–4.960	0.108	0.075
Genital hygiene education/information	2.816	0.884	1.084–4.548	0.142	0.001
Consulting a physician for health problems	2.337	0.999	0.379–4.295	0.094	0.019
Irregular exercise (reference: no exercise)	−0.364	0.935	−2.198–1.469	−0.018	0.697
Regular exercise (reference: no exercise)	0.811	1.413	−1.959–3.580	0.027	0.566
Model summary: R^2^ = 0.196, adjusted R^2^ = 0.178, F = 7.431, *p* < 0.001.					

Note. The dependent variable was the total Genital Hygiene Behaviors Scale score. B = unstandardized regression coefficient; SE = standard error; β = standardized regression coefficient; CI = confidence interval. Reference categories were living in a city, non-smoker/former smoker, never using alcohol, no history of gynecological examination/Pap smear, no genital hygiene education/information, neglecting or self-treating health problems, and no exercise. Age was entered into the regression model as a continuous variable. HC3 robust standard errors were used to account for heteroscedasticity.

## Data Availability

The datasets used and/or analyzed during the current study are available from the corresponding author upon reasonable request.

## Supplementary Materials

The following supporting information can be downloaded at https://www.mdpi.com/article/10.3390/healthcare14183066/s1. Table S1: Item-level descriptive statistics for the HLS and GHBS (*n* = 469).
