# Supplementary material for: Predictors of Genital Hygiene Behaviors Among Women Recruited Through an Online Survey in Türkiye: The Role of Health Literacy
Source: Healthcare (Basel). 2026 Sep 17;14(18):3066. doi: 10.3390/healthcare14183066 (PMC13606843; doi:10.3390/healthcare14183066)
Supplement: Supplementary file 1 [file healthcare-14-03066-s001.zip › healthcare-4536826-supplementary.pdf]

Table S1. Item-level descriptive statistics for the HLS and GHBS (n = 469)

Scale	Item	Reverse-keyed	n	Mean	SD	Median	Q1	Q3	Minimum	Maximum
Health Literacy Scale	HLS item 1	No	469	4.46	0.72	5.00	4.00	5.00	1.00	5.00
Health Literacy Scale	HLS item 2	No	469	4.40	0.74	5.00	4.00	5.00	1.00	5.00
Health Literacy Scale	HLS item 3	No	469	4.60	0.68	5.00	4.00	5.00	1.00	5.00
Health Literacy Scale	HLS item 4	No	469	4.53	0.70	5.00	4.00	5.00	1.00	5.00
Health Literacy Scale	HLS item 5	No	469	4.50	0.73	5.00	4.00	5.00	1.00	5.00
Health Literacy Scale	HLS item 6	No	469	4.34	0.84	5.00	4.00	5.00	1.00	5.00
Health Literacy Scale	HLS item 7	No	469	3.94	1.08	4.00	3.00	5.00	1.00	5.00
Health Literacy Scale	HLS item 8	No	469	4.41	0.83	5.00	4.00	5.00	1.00	5.00
Health Literacy Scale	HLS item 9	No	469	4.71	0.61	5.00	5.00	5.00	1.00	5.00
Health Literacy Scale	HLS item 10	No	469	4.17	0.92	4.00	4.00	5.00	1.00	5.00
Health Literacy Scale	HLS item 11	No	469	4.62	0.65	5.00	4.00	5.00	1.00	5.00
Health Literacy Scale	HLS item 12	No	469	4.62	0.67	5.00	4.00	5.00	1.00	5.00
Health Literacy Scale	HLS item 13	No	469	4.06	1.08	4.00	3.00	5.00	1.00	5.00
Health Literacy Scale	HLS item 14	No	469	4.35	0.83	5.00	4.00	5.00	1.00	5.00
Health Literacy Scale	HLS item 15	No	469	4.20	0.87	4.00	4.00	5.00	1.00	5.00
Health Literacy Scale	HLS item 16	No	469	4.51	0.68	5.00	4.00	5.00	1.00	5.00
Health Literacy Scale	HLS item 17	No	469	4.42	0.77	5.00	4.00	5.00	1.00	5.00
Health Literacy Scale	HLS item 18	No	469	4.46	0.73	5.00	4.00	5.00	1.00	5.00
Health Literacy Scale	HLS item 19	No	469	4.44	0.73	5.00	4.00	5.00	1.00	5.00
Health Literacy Scale	HLS item 20	No	469	4.56	0.68	5.00	4.00	5.00	1.00	5.00
Health Literacy Scale	HLS item 21	No	469	4.29	0.82	4.00	4.00	5.00	1.00	5.00
Health Literacy Scale	HLS item 22	No	469	4.44	0.77	5.00	4.00	5.00	1.00	5.00
Health Literacy Scale	HLS item 23	No	469	4.09	1.01	4.00	4.00	5.00	1.00	5.00
Health Literacy Scale	HLS item 24	No	469	4.17	0.92	4.00	4.00	5.00	1.00	5.00
Health Literacy Scale	HLS item 25	No	469	4.29	0.86	4.00	4.00	5.00	1.00	5.00
Genital Hygiene Behaviors Scale	GHBS item 1	No	469	4.54	1.08	5.00	5.00	5.00	1.00	5.00
Genital Hygiene Behaviors Scale	GHBS item 2	No	469	4.44	0.85	5.00	4.00	5.00	1.00	5.00
Genital Hygiene Behaviors Scale	GHBS item 3	No	469	3.13	1.23	3.00	2.00	4.00	1.00	5.00
Genital Hygiene Behaviors Scale	GHBS item 4	No	469	4.27	0.87	4.00	4.00	5.00	1.00	5.00
Genital Hygiene Behaviors Scale	GHBS item 5	No	469	4.80	0.65	5.00	5.00	5.00	1.00	5.00
Genital Hygiene Behaviors Scale	GHBS item 6	No	469	2.48	1.24	2.00	2.00	3.00	1.00	5.00
Genital Hygiene Behaviors Scale	GHBS item 7	Yes	469	3.73	1.46	4.00	2.00	5.00	1.00	5.00
Genital Hygiene Behaviors Scale	GHBS item 8	No	469	3.56	1.21	4.00	3.00	5.00	1.00	5.00
Genital Hygiene Behaviors Scale	GHBS item 9	No	469	4.46	0.88	5.00	4.00	5.00	1.00	5.00
Genital Hygiene Behaviors Scale	GHBS item 10	No	469	4.74	0.62	5.00	5.00	5.00	1.00	5.00
Genital Hygiene Behaviors Scale	GHBS item 11	No	469	4.83	0.59	5.00	5.00	5.00	1.00	5.00
Genital Hygiene Behaviors Scale	GHBS item 12	No	469	4.72	0.63	5.00	5.00	5.00	1.00	5.00
Genital Hygiene Behaviors Scale	GHBS item 13	No	469	4.68	0.71	5.00	5.00	5.00	1.00	5.00
Genital Hygiene Behaviors Scale	GHBS item 14	Yes	469	4.22	1.21	5.00	4.00	5.00	1.00	5.00
Genital Hygiene Behaviors Scale	GHBS item 15	No	469	3.88	1.21	4.00	3.00	5.00	1.00	5.00
Genital Hygiene Behaviors Scale	GHBS item 16	No	469	4.80	0.57	5.00	5.00	5.00	1.00	5.00
Genital Hygiene Behaviors Scale	GHBS item 17	No	469	4.38	0.94	5.00	4.00	5.00	1.00	5.00

Scale	Item	Reverse-keyed	n	Mean	SD	Median	Q1	Q3	Minimum	Maximum
Genital Hygiene Behaviors Scale	GHBS item 18	No	469	4.50	0.77	5.00	4.00	5.00	1.00	5.00
Genital Hygiene Behaviors Scale	GHBS item 19	Yes	469	3.65	1.32	4.00	3.00	5.00	1.00	5.00
Genital Hygiene Behaviors Scale	GHBS item 20	Yes	469	4.21	1.03	5.00	4.00	5.00	1.00	5.00
Genital Hygiene Behaviors Scale	GHBS item 21	No	469	3.73	1.16	4.00	3.00	5.00	1.00	5.00
Genital Hygiene Behaviors Scale	GHBS item 22	No	469	4.05	1.05	4.00	4.00	5.00	1.00	5.00
Genital Hygiene Behaviors Scale	GHBS item 23	Yes	469	4.16	1.26	5.00	4.00	5.00	1.00	5.00

Note. HLS = Health Literacy Scale; GHBS = Genital Hygiene Behaviors Scale; SD = standard deviation; Q1 and Q3 = 25th and 75th percentiles, respectively.

GHBS item statistics are based on the scored values used to calculate the total score. Items 7, 14, 19, 20, and 23 were reverse-keyed according to the scale scoring instructions.
